# [18F]FDG-PET/CT in *Staphylococcus aureus* bacteremia: a systematic review

**DOI:** 10.1186/s12879-022-07273-x

**Published:** 2022-03-24

**Authors:** D. T. P. Buis, E. Sieswerda, I. J. E. Kouijzer, W. Y. Huynh, G. L. Burchell, M. A. H. Berrevoets, J. M. Prins, K. C. E. Sigaloff

**Affiliations:** 1 Department of Internal Medicine, Division of Infectious Diseases, Amsterdam Institute for Infection and Immunity, Amsterdam UMC, Vrije Universiteit Amsterdam, De Boelelaan 1117, Amsterdam, The Netherlands; 2 Department of Medical Microbiology, University Medical Center Utrecht, Utrecht University, Utrecht, The Netherlands; 3 Julius Center for Health Sciences and Primary Care, University Medical Center Utrecht, Utrecht University, Utrecht, The Netherlands; 4 Radboudumc, Department of Internal Medicine and Radboud Center for Infectious Diseases, Radboud University Medical Center, P.O. Box 9101, 6500 HB Nijmegen, The Netherlands; 5 Medical Library, Vrije Universiteit Amsterdam, De Boelelaan 1117, Amsterdam, The Netherlands; 6 Department of Internal Medicine, Division of Infectious Diseases, Elisabeth TweeSteden Hospital, Tilburg, The Netherlands

**Keywords:** *Staphylococcus aureus*, Bacteremia, [18F]FDG-PET/CT, Systematic review

## Abstract

**Objectives:**

[18F]FDG-PET/CT is used for diagnosing metastatic infections in *Staphylococcus aureus* bacteremia (SAB) and guidance of antibiotic treatment. The impact of [18F]FDG-PET/CT on outcomes remains to be determined. The aim of this systematic review was to summarize the effects of [18F]FDG-PET/CT on all-cause mortality and new diagnostic findingsin SAB.

**Methods:**

We systematically searched PubMed, EMBASE.com, Web of Science, and Wiley’s Cochrane library from inception to 29 January 2021. Eligible studies were randomized controlled trials, clinically controlled trials, prospective and retrospective cohort studies, and case–control studies investigating the effects of [18F]FDG-PET/CT in hospitalized adult patients with SAB. We excluded studies lacking a control group without [18F]FDG-PET/CT. Risk of bias was assessed using the ROBINS-I tool and certainty of evidence using the GRADE approach by two independent reviewers.

**Results:**

We identified 1956 studies, of which five were included in our qualitative synthesis, including a total of 880 SAB patients. All studies were non-randomized and at moderate or serious risk of bias. Four studies, including a total of 804 patients, reported lower mortality in SAB patients that underwent [18F]FDG-PET/CT. One study including 102 patients reported more detected metastatic foci in the participants in whom [18F]FDG-PET/CT was performed.

**Discussion:**

We found low certainty of evidence that [18F]FDG-PET/CT reduces mortality in patients with SAB. This effect is possibly explained by a higher frequency of findings guiding optimal antibiotic treatment and source control interventions.

**Supplementary Information:**

The online version contains supplementary material available at 10.1186/s12879-022-07273-x.

## Introduction

*Staphylococcus aureus* bacteremia (SAB) is one of the most common severe bacterial infections and has a 30-day overall mortality of around 20% [1, 2]. SAB is notorious for causing metastatic infection through hematogenous spread, including endocarditis, osteomyelitis, and abscesses [1, 3]. Risk factors for metastatic infections include community acquisition of bacteremia, a delayed start of adequate antibiotic treatment, positive blood cultures 24 h after start of adequate antibiotic treatment, and persistent fever 72 h after the initial positive blood culture [3, 4].

Accurate and timely diagnosis of metastatic foci of infection is essential in SAB management for multiple reasons. First, current guidelines recommend treating SAB presenting with metastatic infections with a prolonged course of antibiotics, i.e. 4–6 weeks [5]. Moreover, metastatic infections often require specific treatment, and source control of metastatic abscesses is associated with improved outcomes [1, 5]. Early detection of metastatic infections, however, is challenging since patients often do not present with clinical signs or symptoms [6, 7]. Finally, mortality in SAB patients has plateaued over the last decades emphasizing the need for improved diagnostic and therapeutic strategies [1].

2-[^18^F]fluoro-2-deoxy-D-glucose positron emission tomography with combined computed tomography ([18F]FDG-PET/CT) is an imaging modality with potentially broad applications in the field of infectious diseases [8]. Globally, the use of [18F]FDG-PET/CT in the diagnostic workup of infectious diseases varies greatly because of local differences in availability of scanners, costs, and reimbursement for infectious diseases indications [9–11]. For diagnosis of prosthetic valve endocarditis and cardiac implantable electronic device (CIED) infections use of [18F]FDG-PET/CT has been incorporated in international guidelines [12]. In SAB patients, [18F]FDG-PET/CT offers potential for detection of metastatic infections, with subsequent adjustment of antibiotic therapy and source control interventions. Recently, several studies have investigated its use in patients with SAB. In the current study, we performed a systematic review to summarize the effects of [18F]FDG-PET/CT on clinical outcomes in hospitalized adult patients with SAB.

## Methods

### Study design and inclusion criteria

This systematic review was performed according to the Preferred Reporting Items for Systematic Reviews and Meta-Analyses (PRISMA) statement [13]. The study protocol was prospectively registered with PROSPERO (CRD42021238077). We included all randomized controlled trials, clinically controlled trials, prospective and retrospective cohort studies, and case–control studies investigating the effects of [18F]FDG-PET/CT in adult patients with SAB. Case reports, systematic reviews, and meta-analyses were excluded. We also excluded studies lacking a control group without [18F]FDG-PET/CT, studies including less than 20 patients, duplicate studies, and studies without full text available.

Primary outcomes were all-cause mortality rate as defined by the authors and new diagnostic findings on [18F]FDG-PET/CT related to SAB. Secondary outcomes included infection relapse, classification of SAB as complicated or uncomplicated as defined by the authors, change of antibiotic regimen or antibiotic treatment duration, source control interventions, and rate of non-infection related accidental findings on [18F]FDG-PET/CT. Studies which did not investigate at least one of the prespecified outcomes were excluded. In the qualitative synthesis we included only outcome measures for which a formal comparison was made with a group that did not undergo [18F]FDG-PET/CT.

### Search strategy

A systematic search was performed on 29th of January 2021 (by GBL and DTPB), using the databases PubMed, Embase.com, Clarivate Analytics/Web of Science Core Collection and the Wiley/Cochrane Library. The search included keywords and free text terms for (synonyms of) ‘*Staphylococcus aureus*’ combined with (synonyms of) ‘positron emission tomography’. Animal studies were excluded. A full overview of the search terms per database can be found in the supplementary information (see Additional file 1: Appendix SA). No limitations on date or language were applied. The references of the articles included in the qualitative synthesis were searched manually for relevant publications. We manually searched www.clinicaltrials.gov and the International Clinical Trials Registry Platform for ongoing studies. We approached authors of studies that fulfilled all inclusion criteria, but did not report study outcomes separately for SAB patients to obtain these data. Two investigators (DTPB and WYH) independently evaluated all identified studies based on the in- and exclusion criteria as defined in the review protocol. Any discrepancies were resolved by discussion. If no consensus was reached the final decision was made by a third investigator (ES). We used a standardized electronic form for data extraction to collect data concerning study design, population, intervention characteristics, and results. Two investigators (DTPB and WYH) extracted the data using this form and reported independently if data planned to extract was missing. Any discrepancies were resolved by discussion.

### Quality assessment

Two investigators (DTPB and WYH) independently assessed the certainty of the body evidence at the outcome level using the Grading of Recommendations Assessment, Development and Evaluation (GRADE) methodology. Risk of bias of non-randomized studies was assessed at the study level using the ROBINS-I tool by two investigators (DTPB and WYH) independently. In case of discrepancies between the two authors, a third investigator (ES) was consulted. The ROBINS-I is the preferred tool for risk of bias assessment in non-randomized studies according to the Cochrane Collaboration [14]. This tool distinguishes seven different bias domains: bias due to confounding, bias in selection of participants, bias in classification of intervention, bias due to deviations from intended interventions, bias due to missing data, bias in measurement of the outcome, and bias in selection of the reported result. The full methods of our risk of bias assessment is set out in Additional file 2: Appendix SB. We did not assess publication bias with funnel plot inspection, because of the limited number of studies included in the qualitative synthesis.

### Quantitative synthesis

We planned to perform a meta-analysis including sufficiently homogenous studies with regard to study design and study outcomes, i.e. prospective studies with a control group, performance of [18F]FDG-PET/CT within 14 days of diagnosis of SAB, and similar reporting of study outcomes. Since only one study fulfilled these criteria we did not perform a quantitative synthesis [15].

## Results

### Search results

**Fig. 1:**
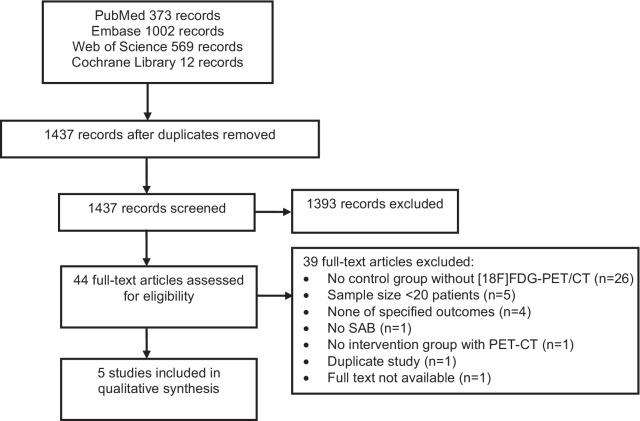
PRISMA flow diagram of study selection for the review

We identified 1956 records through database searches: 373 through PubMed, 1002 through Embase, 569 through Web of Science, and 12 through the Cochrane Library (Fig. 1). After removal of duplicates we screened 1437 records and assessed 44 full text articles for eligibility. We included five studies in our final qualitative synthesis. The most common reason for exclusion during the full text assessment was lack of a control group without [18F]FDG-PET/CT (n = 26). Review of references of articles included in the qualitative syntheses did not result in additional studies. We retrieved one relevant ongoing study via manual searching of www.clinicaltrials.gov and the International Clinical Trials Registry Platform (ICTRP) [16]. We obtained data on 3 month mortality in SAB patients by approaching the authors of one of the studies [17].

### Included studies

**Table ****1** displays the characteristics of the five included studies, including a total of 880 patients [15, 17–20]. Three of the studies were performed in The Netherlands, one in Belgium and one in Israel. All included studies were non-randomized. 3 studies were retrospective cohort studies, one a prospective cohort study, and one a prospective cohort study with historical controls. Prevalence of methicillin resistant *Staphylococcus aureus* varied between 0 and 22%. Three studies only included SAB patients with risk factors for metastatic infections. Risk factors in these studies included community acquisition, signs of infection more than 48 h before initiation of appropriate treatment, fever more than 72 h after initiation of appropriate treatment, positive blood cultures more than 48 h after initiation of appropriate treatment or presence of foreign body materials. In the other two studies the prevalence of risk factors was 46 and 80%, respectively. In all five studies, median or mean time between diagnosis of SAB and performance of [18F]FDG-PET/CT was 11 days or less (range 7–11 days). Follow-up duration ranged between 3 months and one year.

**Table 1:** Study characteristics

First author, year	Country	Enrolment period	Design	Population	n SAB patients	% MRSA	% Persistent bacteremia	Timing PET-CT	Additional diagnostic procedures^1^	Outcomes
Vos 2010 [17]	The Netherlands	Prospective cohort: 2005–2008 Historical control group: 2000–2004	Prospective cohort study with historical controls	Prospective cohort: Adult patients with high-risk SAB who received PET-CT Historical control group: Adult patients with high-risk SAB who did not receive PET-CT	Prospective cohort: 73 Historical controls: 146	Unknown	NR	Median 7 days. Mean 6.8 days Maximum 14 days For SAB specific unknown	NR	3 month infection relapse, 3 month mortality
Berrevoets 2017 [19]	The Netherlands	2013–2016	Retrospective cohort study	Consecutive SAB cases. In intervention group 99/105 patients with high-risk bacteremia	Intervention: 105 Control: 79	2.7%	Intervention: 33% Control: 16%	Median 8.0 days Mean 8.7 days	Only reported in patients with high-risk bacteremia Echocardiography in 20 patients (41%) in intervention group and 90 (91%) patients in control group with high-risk bacteremia	3 month mortality, 3 month infection relapse in high-risk SAB subgroup
Berrevoets 2019 [20]	The Netherlands	2013–2017	Retrospective cohort study	Cases: High-risk SAB with no metastatic infections on PET-CT and normal echocardiography Controls: SAB without risk factors and no known metastatic disease	Cases: 36 Controls: 40	0%	Cases: 17% Controls: 0%	Mean 8.4 days	Echocardiography in 36 cases (100%) and 10 controls (25%)	3 month mortality, 3 month SAB specific mortality, 3 month infection relapse
Yildiz 2019 [18]	Belgium	2014–2017	Retrospective cohort study	Adult patients with high-risk SAB	Intervention: 48 Control: 54	5.9%	NR	Within 7 days	TTE in all patients in intervention and control group. TEE in 47 patients (98%) in intervention group and 41 (76%) in control group	1 month mortality, 3 month mortality, one-year mortality, new diagnostic findings related to SAB
Ghanem-Zoubi 2020 [15]	Israel	2015–2019	Prospective cohort study	Adult patients with SAB	Intervention: 149 Control: 150	Intervention: 23% Control: 22%	Intervention: 29% Control: 17%	Median 11 days	TEE in 133 patients (89%)in intervention group and 90 (61%) in control group	1 month mortality, 3 month mortality, 6 month mortality, 6 month infection relapse, any intervention after bacteremia, duration of appropriate antibiotic treatment

*NR* not reported; PET-CT, [18F]FDG-PET/CT; *SAB* *Staphylococcus aureus* bacteremia; *TTE* transthoracic echocardiography; *TEE* transesophageal echocardiography

^1^Performance of conventional radiological techniques was reported in none of the studies

### Primary outcomes

**Fig. 2:**
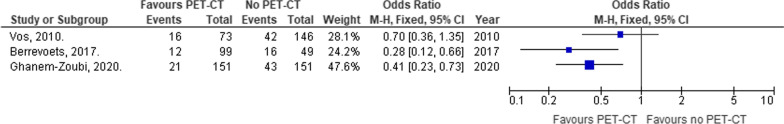
Effect of [18F]FDG-PET/CT vs. no [18F]FDG-PET/CT on 3-month all-cause mortality in patients with SAB. PET-CT, [18F]FDG-PET/CT; SAB, *Staphylococcus aureus* bacteremia

The main results of included studies are shown in Table 2. Four studies compared 3-month mortality rates between an intervention group who underwent [18F]FDG-PET/CT and a control group who did not[15, 17–19]. All four reported lower 3-month mortality rates in the intervention group. Three of these studies reported respectively a 7, 15 and 21% reduction in mortality and one did not provide an effect estimate [15–18]. The results of these studies are depicted in Fig. 2. Moreover, three of these studies observed an association between performance of [18F]FDG-PET/CT and lower mortality in multivariate regression analyses with adjustment for confounding variables. Two of these three studies also performed analyses with mortality at other time points as outcome, i.e. 1, 6-month and one-year mortality, which all showed consistent lower mortality rates in the intervention group [15, 18].

**Table 2:** Main results included studies

First author, year	1 month mortality	3 month mortality	3 month SAB-specific mortality	6 month mortality	1-year mortality	New SAB-related diagnostic findings	Infection relapse rate	Duration of appropriate antibiotic treatment	Performance of any intervention after bacteremia
Vos 2010 [17]	NR	With PET-CT: 21.9% Without PET-CT: 28.8% p = 0.18 OR 0.7 (0.36; 1.35)	NR	NR	NR	NR	With PET-CT: 1.4% Without PET-CT: 8.9% p = 0.04	NR	NR
Berrevoets 2017 [19]	NR	With PET-CT: 12.1% Without PET-CT: 32.7% p = 0.003 OR 0.28 (0.12; 0.66)	NR	NR	NR	NR	In high risk SAB subgroup: 0% with PET-CT and 3% without PET-CT	NR	NR
Berrevoets 2019 [20]	NR	Cases: 19.4% Controls: 15.0% p = 0.64 OR 1.37 (0.41; 4.53)	Cases: 0% Controls: 2.5% p = 1.00	NR	NR	NR	Cases: 2.8% Controls: 5.0% p = 1.00	NR	NR
Yildiz 2019 [18]	No estimate provided p = 0.001	No estimate provided p = 0.004	NR	NR	With PET-CT: 16.6% Without PET-CT: 44.4% p = 0.002	49 foci with PET-CT 13 foci without PET-CT p < 0.00001	NR	NR	NR
Ghanem-Zoubi 2020 [15]	With PET-CT: 4% Without PET-CT: 13% p = 0.004	With PET-CT: 14% Without PET-CT: 29% p = 0.002 OR 0.41 (0.23; 0.73)	NR	With PET-CT: 23% Without PET-CT: 35% p = 0.023	NR	NR	With PET-CT: 3.3% Without PET-CT: 2.6% p = 0.735	With PET-CT: 42 days Without PET-CT: 19 days p = 0.001	With PET-CT: 22% Without PET-CT: 12% p = 0.021

*NR* not reported; PET-CT, [18F]FDG-PET/CT; *OR* odds ratio. With 95% confidence interval

One study applied a different approach, comparing mortality between two groups with different characteristics [20]. The authors retrospectively compared patient outcomes between cases *with* risk factors for metastatic infections but *without* signs of metastatic infection on [18F]FDG-PET/CT and normal echocardiography with controls *without* these risk factors and no known metastatic disease. This control group did not undergo [18F]FDG-PET/CT. This study showed no statistically significant difference in mortality (19.4 vs. 15.0%, p = 0.64) and SAB-specific mortality (0 vs. 2.5%, p = 1.00) between the two groups [20].

Three of the included studies reported new diagnostic findings related to SAB as an outcome measure. However, only one study compared this outcome between the intervention and the control group and reported more detected metastatic foci in the participants who underwent [18F]FDG-PET/CT. This study reported 49 diagnostic foci detected in 48 patients in the PET-CT group and 13 foci in 54 patients in the control group (p < 0.00001) [18]. The other two studies only reported the rate of new findings (in 71 and 70% of patients, respectively) in the [18F]FDG-PET/CT group, but not in the control group [15, 19]. Additional file 3: Table S1 describes diagnostic findings on [18F]FDG-PET/CT per organ system in individual studies.

### Secondary outcomes

Four studies reported on risk of infection relapse as outcome [15, 17, 19, 20]. One study found a lower cumulative incidence of relapse in the intervention group (1.4% vs. 8.9%, p = 0.04) and two studies found no difference between both groups (3 vs. 3%, p = 0.74; 0 vs. 3%, p = 0.56) [15, 17, 19]. The study that compared cases with risk factors for complicated bacteremia without signs of metastatic infection on [18F]FDG-PET/CT and normal echocardiography with controls without risk factors and no known metastatic disease showed no statistically significant difference in cumulative incidence of infection relapse (2.8 vs. 5.0%, p = 1.00 [20].

One study compared duration of appropriate antibiotic treatment between the intervention and the control group and reported a higher (42 vs. 19 days, p = 0.001) duration of appropriate antibiotic treatment in the group that underwent [18F]FDG-PET/CT [15]. One study reported more interventions in patients who received [18F]FDG-PET/CT (22 vs. 12%, p = 0.021), but it was unclear whether these interventions included only source control interventions specifically for SAB and which proportion was performed after [18F]FDG-PET/CT [15]. Only one study reported non-infection related accidental findings on [18F]FDG-PET/CT, which were detected in 7.1% of the patients [19]. None of the studies reported final disease classification of complicated versus uncomplicated SAB as a separate outcome.

### Risk of bias assessment

**Fig. 3:**
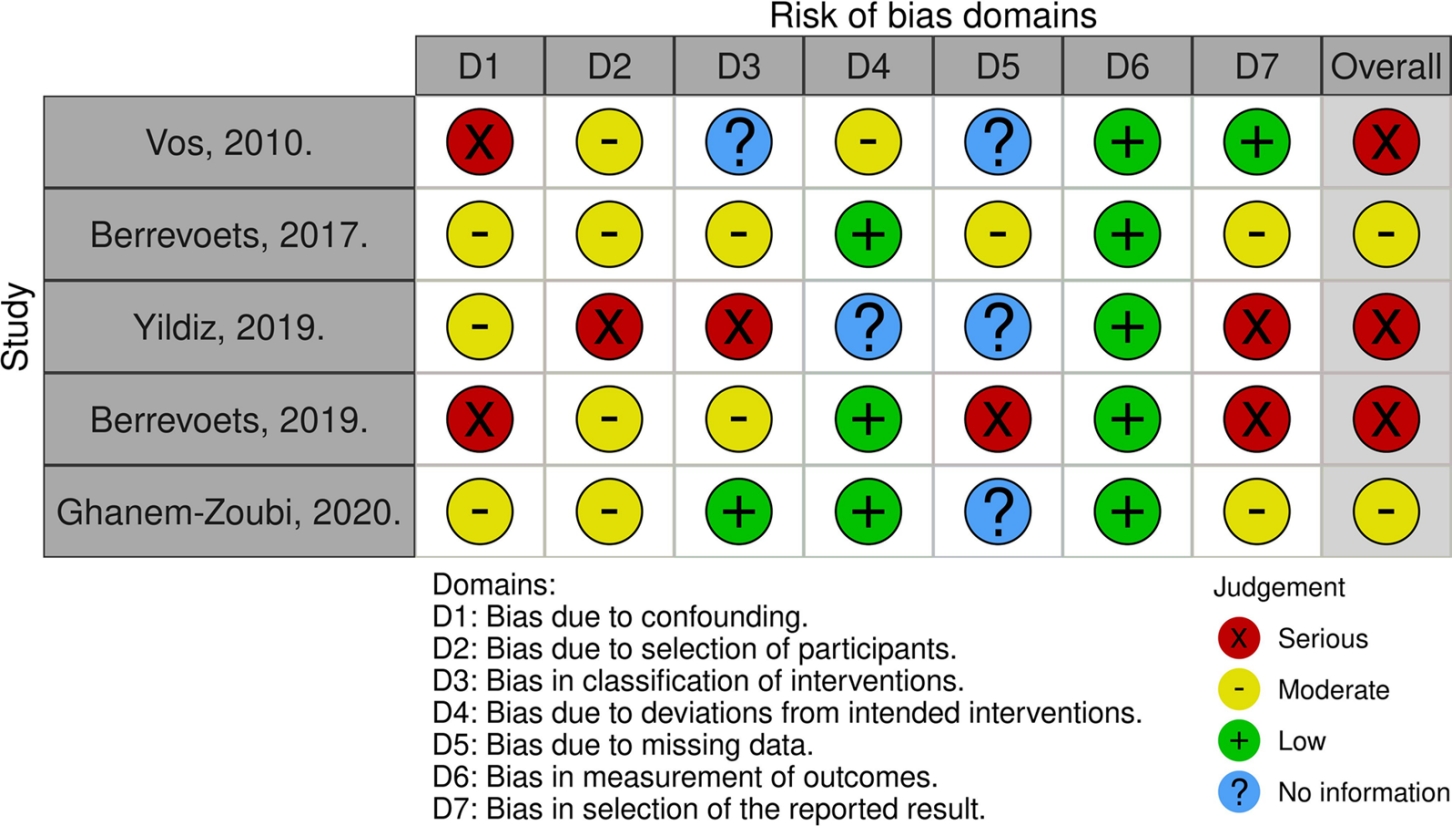
Risk of bias according the ROBINS-I tool

We identified three important potential confounding domains for studies included in our review: demographics and comorbidities of the study population, severity of disease, and use of co-interventions. Using ROBINS-I assessment, we judged three studies to be at overall serious risk of bias and two studies at overall moderate risk of bias, as shown in Fig. 3. The main reasons for judgment of high risk of bias were bias due to confounding and bias in selection of the reported result. Two studies used matching and two performed multivariate regression analyses to adjust for confounding variables. One study did not employ methods to avoid confounding or adjust for it. Additional file 4: Appendix SC displays the risk of bias assessment per domain for the individual studies.

### GRADE assessment

Table 3 shows the GRADE assessments of certainty of evidence for the primary and secondary outcomes. Certainty of evidence for all outcomes ranged from very low to low. For the primary outcomes of our review, we found low certainty evidence that [18F]FDG-PET/CT is associated with lower mortality and very low certainty evidence that it leads to more new SAB-related diagnostic findings. Most common reasons for downgrading of level of evidence were risk of bias and imprecision of the effect estimates.

**Table 3:** GRADE assessment quality of evidence

[18F]FDG-PET/CT vs. no [18F]FDG-PET/CT in hospitalized adult patients with *Staphylococcus aureus* bacteremia Population: Hospitalized adult patients with *Staphylococcus aureus* bacteremia Intervention: ^18^F-FDG PET/CT Comparison: No ^18^F-FDG PET/CT		
**Outcome**	**N participants, n studies**	**Quality of the evidence (GRADE)**
Mortality	880 participants, 5 studies	Low
New diagnostic findings detected by PET-CT	102 participants, 1 study	Very low
Infection relapse rate	778 participants, 4 studies	Very low
SAB-specific mortality	76 participants, 1 study	Very low
Change of antibiotic treatment duration and regimen	299 participants, 1 study	Very low
Performance of source control interventions	299 participants, 1 study	Very low

GRADE Working Group grades of evidence

High quality: The authors have a lot of confidence that the true effect is similar to the estimated effect

Moderate quality: The authors believe that the true effect is probably close to the estimated effect

Low quality: The true effect might be markedly different from the estimated effect

Very low quality: The true effect is probably markedly different from the estimated effect

## Discussion

In our systematic review we found low certainty evidence that [18F]FDG-PET/CT is associated with lower mortality and very low certainty evidence that it leads to more SAB-related diagnostic findings in patients with SAB. A limited number of studies investigated this research question and all included studies were non-randomized, which makes them inherently at risk of bias. Nevertheless, the studies fairly consistently showed that performing [18F]FDG-PET/CT is associated with a large reduction in mortality.

Since ^18^F-FDG PET/CT is an imaging modality without therapeutic effects, its association with improved survival in SAB must be mediated by therapeutic interventions, especially modifications in antibiotic treatment and source control interventions. Up to a third of patients with gram-positive bacteremia and metastatic infection does not have guiding signs or symptoms [7]. Therefore, a diagnostic work-up strategy guided by clinical presentation alone is at high risk for missing relevant metastatic infections. Our review yielded only one study that reported on these treatment modifications following [18F]FDG-PET/CT as compared to patients that did not undergo [18F]FDG-PET/CT [15]. Based on this study, there is very low certainty evidence for a longer duration of appropriate antibiotic treatment (42 vs. 19 days) and more frequent performance of interventions (22 vs. 12%) in patients undergoing [18F]FDG-PET/CT [15]. Other studies in our review also reported a high incidence of new SAB-related diagnostic findings and subsequent treatment modifications in patients undergoing ^18^F-FDG PET/CT [18, 20]. However, these latter studies did not report on these outcomes in the control group without [18F]FDG-PET/CT, precluding a comparison between groups.

An important question for clinical practice is which subgroup of patients would benefit most from performing [18F]FDG-PET/CT. SAB is a very heterogeneous disease and its clinical course can range from mild to extremely severe, which warrants patient tailored clinical management [1]. A strategy of performing [18F]FDG-PET/CT in all patients with SAB is at risk for over-testing, unnecessary radiation burden, and expenditure of scarce recourses. In clinical practice, [18F]FDG-PET/CT is most often performed in patients with risk factors for metastatic infections, since these patients are supposed to have a higher prior probability of finding relevant findings. Most studies in our review included patients with a high frequency of these risk factors in the intervention group. Therefore we were not able to identify which subgroups of SAB patients would benefit most from [18F]FDG-PET/CT. Scarce evidence exists to support its use in patients without risk factors for metastatic infection. One study reported a stronger association between [18F]FDG-PET/CT and lower mortality in low-risk SAB than in high-risk SAB (unadjusted OR 0.27 (95% CI 0.10–0.72) versus 0.44 (95% CI 0.20–0.98)) [15]. The group of 67 patients with low-risk SAB had a comparable frequency of SAB-related diagnostic findings (61.2%) as the 84 patients with high-risk SAB (66.7%). Further studies must investigate whether this beneficial effect of [18F]FDG-PET/CT in low-risk SAB is robust.

Besides diagnosing metastatic infections that generally warrant prolongation of antibiotic treatment, [18F]FDG-PET/CT also has the potential to justify shorter antibiotic treatment in selected patients. The Infectious Diseases Society of America (IDSA) guideline states that patients with positive follow-up blood cultures performed 48–96 h after the initial culture and persistent fever after 72 h of adequate antibiotic therapy must be classified as having complicated bacteremia and should be treated with an extended antibiotic course, i.e. 4–6 weeks [5]. One study in our review showed that patients with risk factors for metastatic infections but normal [18F]FDG-PET/CT and echocardiography results received similar antibiotic treatment duration and had similar outcomes as patients with uncomplicated bacteremia who did not undergo [18F]FDG-PET/CT [20]. This finding suggests that these patients could be “reclassified” as having uncomplicated bacteremia by performing ^18^F-FDG PET/CT and possibly be treated with a shorter course of antibiotics. However, this study was underpowered to detect a statistically significant difference between both groups. Moreover, certain risk factors yield higher risk of metastatic complications than others, making it unclear whether this strategy is equally safe in all patients in this subgroup [3].

Strengths of our systematic review include the prospectively registered study protocol, rigorous systematic bibliographic search, and extensive assessment of bias using the ROBINS-I tool. Our study also has several important limitations. First, only a limited number of studies were identified which addressed the research question and three out of five studies were performed in The Netherlands [17, 19, 20]. Second, performing a meaningful meta-analysis was not possible since studies were not sufficiently homogeneous. Lastly, all included studies were non-randomized and therefore prone to bias, especially confounding by indication and immortal time bias. Confounding by indication could lead to a biased effect estimate if [18F]FDG-PET/CT was less likely to be performed in patients with certain characteristics. For example, not performing [18F]FDG-PET/CT in patients with severe disease because they were too sick to undergo [18F]FDG-PET/CT would lead to a bias towards [18F]FDG-PET/CT being beneficial. Another important potential form of bias in the included studies was immortal time bias. Immortal time bias could occur by including patients that died before [18F]FDG-PET/CT could be performed. These patients would be classified in the group that did not receive [18F]FDG-PET/CT, leading to a biased effect estimate in favor of undergoing PET-CT.

The findings of our systematic review warrant further research directed at the effects of performing [18F]FDG-PET/CT in SAB. Ultimately, a randomized controlled trial (RCT) could provide higher-quality evidence. Currently, a RCT is being performed in France which randomizes adult SAB patients without infective endocarditis to [18F]FDG-PET/CT or to a control group with routine care 14 days after the SAB diagnosis [16]. Primary outcome is presence of deep foci of infection and secondary outcomes include 3- and 6 month survival and cost-effectiveness. This study, however, does not use frequency and clinical consequences of non-infection related accidental findings on [18F]FDG-PET/CT as an outcome, which is necessary to enable an informed cost–benefit analysis. Non-infection related accidental findings could lead to unnecessary and potentially harmful diagnostic and therapeutic interventions. Other potential disadvantages of [18F]FDG-PET/CT include the harmful effects of radiation and the associated monetary costs.

In summary, our systematic review showed that based on limited evidence of very low to low certainty, [18F]FDG-PET/CT leads to lower mortality in patients with SAB and a higher frequency of SAB-related diagnostic findings. Its effect on other clinical outcomes is yet unclear. Future studies should further define subgroups of SAB patients that benefit most from [18F]FDG-PET/CT.

## Supplementary Information

**Additional file 1. Appendix SA.** Search strategy review.

**Additional file 2. Appendix SB.** Methods risk of bias assessment.

**Additional file 3. Table S1.** Diagnostic findings on [18F]FDG-PET/CT per study.

**Additional file 4. Appendix SC.** Results risk of bias assessment individual studies with ROBINS-I.

## Data Availability

The datasets used and/or analyzed during the current study are available from the corresponding author on reasonable request.

## References

[1] Tong SYC, Davis JS, Eichenberger E, Holland TL, Fowler VG (2015). *Staphylococcus aureus* infections: epidemiology, pathophysiology, clinical manifestations, and management. Clin Microbiol Rev.

[2] van Hal SJ, Jensen SO, Vaska VL, Espedido BA, Paterson DL, Gosbell IB (2012). Predictors of mortality in *Staphylococcus aureus* Bacteremia. Clin Microbiol Rev.

[3] Fowler VG, Olsen MK, Corey GR, Woods CW, Cabell CH, Reller LB (2003). Clinical identifiers of complicated *Staphylococcus aureus* bacteremia. Arch Intern Med.

[4] Kuehl R, Morata L, Boeing C, Subirana I, Seifert H, Rieg S (2020). Defining persistent *Staphylococcus aureus* bacteraemia: secondary analysis of a prospective cohort study. Lancet Infect Dis.

[5] Liu C, Bayer A, Cosgrove SE, Daum RS, Fridkin SK, Gorwitz RJ (2011). Clinical practice guidelines by the infectious diseases society of america for the treatment of methicillin-resistant Staphylococcus aureus infections in adults and children. Clin Infect Dis.

[6] Ringberg H, Thorén A, Lilja B (2000). Metastatic complications of *Staphylococcus aureus* septicemia. To seek is to find Infection.

[7] Cuijpers ML, Vos FJ, Bleeker-Rovers CP, Krabbe PF, Pickkers P, van Dijk AP (2007). Complicating infectious foci in patients with *Staphylococcus aureus* or Streptococcus species bacteraemia. Eur J Clin Microbiol Infect Dis.

[8] Kung BT, Seraj SM, Zadeh MZ, Rojulpote C, Kothekar E, Ayubcha C (2019). An update on the role of (18)F-FDG-PET/CT in major infectious and inflammatory diseases. Am J Nucl Med Mol Imaging.

[9] Douglas AP, Thursky KA, Worth LJ, Harrison SJ, Hicks RJ, Slavin MA (2019). Access, knowledge and experience with fluorodeoxyglucose positron emission tomography/computed tomography in infection management: a survey of Australia and New Zealand infectious diseases physicians and microbiologists. Intern Med J.

[10] Rahman WT, Wale DJ, Viglianti BL, Townsend DM, Manganaro MS, Gross MD (2019). The impact of infection and inflammation in oncologic (18)F-FDG PET/CT imaging. Biomed Pharmacother..

[11] Li Y, Wang Q, Wang X, Li X, Wu H, Wang Q (2020). Expert Consensus on clinical application of FDG PET/CT in infection and inflammation. Ann Nucl Med.

[12] Habib G, Lancellotti P, Antunes MJ, Bongiorni MG, Casalta J-P, Del Zotti F (2015). 2015 ESC guidelines for the management of infective endocarditis: the task force for the management of infective Endocarditis of the European Society of Cardiology (ESC)Endorsed by: European Association for Cardio-Thoracic Surgery (EACTS), the European Association of Nuclear Medicine (EANM). Eur Heart J.

[13] Liberati A, Altman DG, Tetzlaff J, Mulrow C, Gøtzsche PC, Ioannidis JP (2009). The PRISMA statement for reporting systematic reviews and meta-analyses of studies that evaluate healthcare interventions: explanation and elaboration. BMJ.

[14] Higgins JPT, Thomas J, Chandler J, Cumpston M, Tianjing L, Page MJ (2019). Cochrane handbook for systematic reviews of interventions.

[15] Ghanem-Zoubi N, Kagna O, Abu-Elhija J, Mustafa-Hellou M, Qasum M, Keidar Z (2020). Integration of FDG-PET/CT in the diagnostic workup for *Staphylococcus aureus* bacteremia: a prospective interventional matched-cohort study. Clin Infect Dis.

[16] Impact of 18 FDG PET/CT on the management of patients with Staphylococcus aureus bloodstream infection. https://ClinicalTrials.gov/show/NCT03419221.

[17] Vos FJ, Bleeker-Rovers CP, Sturm PD, Krabbe PF, van Dijk AP, Cuijpers ML (2010). 18F-FDG PET/CT for detection of metastatic infection in gram-positive bacteremia. J Nucl Med.

[18] Yildiz H, Reychler G, Rodriguez-Villalobos H, Orioli L, D'Abadie P, Vandeleene B (2019). Mortality in patients with high risk *Staphylococcus aureus* bacteremia undergoing or not PET-CT: a single center experience. J Infect Chemother.

[19] Berrevoets MAH, Kouijzer IJE, Aarntzen E, Janssen MJR, De Geus-Oei LF, Wertheim HFL (2017). (18)F-FDG PET/CT optimizes treatment in *Staphylococcus aureus* bacteremia and is associated with reduced mortality. J Nucl Med.

[20] Berrevoets MAH, Kouijzer IJE, Slieker K, Aarntzen E, Kullberg BJ, Oever JT (2019). (18)F-FDG PET/CT-guided treatment duration in patients with high-risk *Staphylococcus Aureus* bacteremia: a proof of principle. J Nuclear Med.

