# Supplementary material for: [18F]FDG-PET/CT in Staphylococcus aureus bacteremia: a systematic review
Source: BMC Infect Dis. 2022 Mar 24;22:282. doi: 10.1186/s12879-022-07273-x (PMC8943998; doi:10.1186/s12879-022-07273-x)
Supplement: Supplementary file 1 — Additional file 1. Appendix SA. Search strategy review. [file 12879_2022_7273_MOESM1_ESM.docx]

# Additional file 1: Appendix SA: Search strategy review.

## Appendix table 1: Search strategy in PubMed

| **Search** | **Query** | **Results** |
| --- | --- | --- |
| **#8** | **#7 NOT ("Animals"[Mesh] NOT "Humans"[Mesh])** | **373** |
| **#7** | **#3 AND #6** | **471** |
| **#6** | **#4 OR #5** | **209,571** |
| **#5** | "Tomography, Emission-Computed"[Mesh] OR "spect"[tiab] OR "petscan*"[tiab] OR "pet"[tiab] OR ("emission"[tiab] AND "tomograph*"[tiab]) OR "radionuclid*"[tiab] | **209,564** |
| **#4** | **("Deoxyglucose"[Mesh] OR "deoxyglucose"[tw] OR "desoxyglucose"[tw] OR "deoxy-glucose"[tw] OR "desoxy-glucose"[tw] OR "deoxy-d-glucose"[tw] OR "desoxy-d-glucose"[tw] OR "2deoxyglucose"[tw] OR "2deoxy-d-glucose"[tw] OR "fluorodeoxyglucose"[tw] OR "fluorodesoxyglucose"[tw] OR "fludeoxyglucose"[tw] OR "fluordeoxyglucose"[tw] OR "fluordesoxyglucose"[tw] OR "18fluorodeoxyglucose"[tw] OR "18fluorodesoxyglucose"[tw] OR "18fluordeoxyglucose"[tw] OR "fdg"[tw] OR "18fdg*"[tw] OR "18f-dg*"[tw] OR "18f-fdg*"[tw] OR "fdg18*"[tw] OR "fdgpet*"[tw] OR (("fluor"[tw] OR "2fluor*"[tw] OR "fluoro"[tw] OR "fluorodeoxy"[tw] OR "fludeoxy"[tw] OR "fluorine"[tw] OR "18f"[tw] OR "18flu*"[tw]) AND ("glucose"[tw] OR "galactose"[tw]))) AND ("Tomography, Emission-Computed"[Mesh] OR "pet"[tw] OR "pet/ct"[tw] OR "petct"[tw] OR "petscan*"[tw] OR "fdgpet*"[tw] OR ("emission"[tw] AND ("tomogra*"[tw])))** | **46,324** |
| **#3** | #1 OR #2 | **133,531** |
| **#2** | ("Bacteremia"[Mesh] OR "bacteremia*"[tiab] OR "bacteraemia*"[tiab]) AND ("Gram-Positive Bacteria"[Mesh] OR "Gram-Positive Bacter*"[tiab]) | **12,134** |
| **#1** | **"Staphylococcus aureus"[Mesh] OR "Staphylococcus aureus"[tiab]** | **125,844** |

## Appendix table 2: Search strategy in Embase.com

| **Search** | **Query** | **Results** |
| --- | --- | --- |
| **#8** | #7 NOT ([animals]/lim NOT [humans]/lim) | **1,002** |
| **#7** | #3 AND #6 | **1,209** |
| **#6** | #4 OR #5 | **367,011** |
| **#5** | 'computer assisted emission tomography'/exp OR 'gated single photon emission computed tomography'/exp OR 'single photon emission computer tomography'/exp OR petscan*:ti,ab,kw OR pet:ti,ab,kw OR ((emission NEAR/3 tomograph*):ti,ab,kw) OR radionuclid*:ti,ab,kw | **366,827** |
| **#4** | ('deoxyglucose'/exp OR 'deoxyglucose':ab,ti,kw OR 'desoxyglucose':ab,ti,kw OR 'deoxy glucose':ab,ti,kw OR 'desoxy glucose':ab,ti,kw OR 'deoxy d glucose':ab,ti,kw OR 'desoxy d glucose':ab,ti,kw OR '2deoxyglucose':ab,ti,kw OR '2deoxy d glucose':ab,ti,kw OR 'fluorodeoxyglucose':ab,ti,kw OR 'fluorodesoxyglucose':ab,ti,kw OR 'fludeoxyglucose':ab,ti,kw OR 'fluordeoxyglucose':ab,ti,kw OR 'fluordesoxyglucose':ab,ti,kw OR '18fluorodeoxyglucose':ab,ti,kw OR '18fluorodesoxyglucose':ab,ti,kw OR '18fluordeoxyglucose':ab,ti,kw OR 'fdg*':ab,ti,kw OR '18fdg*':ab,ti,kw OR '18f dg*':ab,ti,kw OR '18f fdg*':ab,ti,kw OR 'fdgpet*':ab,ti,kw OR (('fluor':ab,ti,kw OR '2fluor*':ab,ti,kw OR 'fluoro':ab,ti,kw OR 'fluorodeoxy':ab,ti,kw OR 'fludeoxy':ab,ti,kw OR 'fluorine':ab,ti,kw OR '18f':ab,ti,kw OR '18flu*':ab,ti,kw) AND ('glucose':ab,ti,kw OR 'galactose':ab,ti,kw))) AND ('positron emission tomography'/exp OR 'pet*':ab,ti,kw OR 'petscan*':ab,ti,kw OR 'fdgpet*':ab,ti,kw OR ('emission':ab,ti,kw AND 'tomogra*':ab,ti,kw)) | **72,438** |
| **#3** | #1 OR #2 | **214,203** |
| **#2** | ('bacteremia'/exp OR 'bacteremia*':ti,ab,kw OR 'bacteraemia*':ti,ab,kw) AND ('gram positive bacterium'/exp OR 'gram positive bacter*':ti,ab,kw) | **20,325** |
| **#1** | 'staphylococcus aureus'/exp OR 'staphylococcus aureus':ti,ab,kw | **203,061** |

## Appendix table 3: Search strategy in Clarivate Analytics/Web of Science Core Collection

| **Search** | **Query** | **Results** |
| --- | --- | --- |
| **#9** | #7 NOT #8 | **569** |
| **#8** | WC=Agriculture, Dairy & Animal Science OR WC=Veterinary Sciences | **961,164** |
| **#7** | #6 AND #3 | **634** |
| **#6** | #5 OR #4 | **330,965** |
| **#5** | TS=(“spect” OR “petscan*” OR “pet” OR (“emission” AND “tomograph*”) OR “radionuclid*”) | **316,379** |
| **#4** | TS=(“deoxyglucose” OR “desoxyglucose” OR “deoxy glucose” OR “desoxy glucose” OR “deoxy d glucose” OR “desoxy d glucose” OR “2deoxyglucose” OR “2deoxy d glucose” OR “fluorodeoxyglucose” OR “fluorodesoxyglucose” OR “fludeoxyglucose” OR “fluordeoxyglucose” OR “fluordesoxyglucose” OR “18fluorodeoxyglucose” OR “18fluorodesoxyglucose” OR “18fluordeoxyglucose” OR “fdg*” OR “18fdg*” OR “18f dg*” OR “18f fdg*” OR “fdgpet*” OR (“fluor” OR “2fluor*” OR “fluoro” OR “fluorodeoxy” OR “fludeoxy” OR “fluorine” OR “18f” OR “18flu*” AND (“glucose” OR “galactose”)) AND (“pet*” OR “petscan*” OR “fdgpet*” OR (“emission” AND (“tomogra*”)))) | **79,906** |
| **#3** | #2 OR #1 | **138,989** |
| **#2** | TS=(("bacteremia*" OR "bacteraemia*") AND ("Gram-Positive Bacter*") ) | **964** |
| **#1** | TS=("Staphylococcus aureus") | **138,420** |

## Appendix table 4: Search strategy in Wiley/Cochrane Library

| **Search** | **Query** | **Results** |
| --- | --- | --- |
| **#7** | #6 AND #3 | **12** |
| **#6** | #5 OR #4 | **12,941** |
| **#5** | (spect OR petscan* OR pet OR (emission AND tomograph*) OR radionuclid*):ti,ab,kw | **12,784** |
| **#4** | (deoxyglucose OR desoxyglucose OR (deoxy NEAR glucose) OR (desoxy NEAR glucose) OR (deoxy NEAR d NEAR glucose) OR (desoxy NEAR d NEAR glucose) OR 2deoxyglucose OR (2deoxy NEAR d NEAR glucose) OR fluorodeoxyglucose OR fluorodesoxyglucose OR fludeoxyglucose OR fluordeoxyglucose OR fluordesoxyglucose OR 18fluorodeoxyglucose OR 18fluorodesoxyglucose OR 18fluordeoxyglucose OR fdg* OR 18fdg* OR (18f NEAR dg*) OR (18f NEAR fdg*) OR fdgpet* OR ((fluor OR 2fluor* OR fluoro OR fluorodeoxy OR fludeoxy OR fluorine OR 18f OR 18flu*) AND (glucose OR galactose)) AND (pet* OR petscan* OR fdgpet* OR (emission AND (tomogra*)))):ti,ab,kw | **2,608** |
| **#3** | #2 OR #1 | **3,598** |
| **#2** | ((bacteremia* OR bacteraemia*) AND (Gram NEAR Positive NEAR Bacter*)):ti,ab,kw | **180** |
| **#1** | (Staphylococcus NEAR aureus):ti,ab,kw | **3,455** |
