# Supplementary material for: [18F]FDG-PET/CT in Staphylococcus aureus bacteremia: a systematic review
Source: BMC Infect Dis. 2022 Mar 24;22:282. doi: 10.1186/s12879-022-07273-x (PMC8943998; doi:10.1186/s12879-022-07273-x)
Supplement: Supplementary file 2 — Additional file 2. Appendix SB. Methods risk of bias assessment. [file 12879_2022_7273_MOESM2_ESM.docx]

**Additional file 2: Appendix SB: Methods risk of bias assessment.**

**Target trial:**

Design: individually randomized

Participants: Hospitalized adult patients with *S. aureus* bacteremia of any cause defined as at least one positive blood culture with *S. aureus*.
Intervention: Performance of ^18^ F-FDG PET/CT.
Control: No performance of ^18^ F-FDG PET/CT.

The aim of this study is to assess the effect of assignment to the intervention.

The primary and secondary outcomes were considered to be sufficiently similar to have similar confounding variables.

Based on discussion with the review team we identified the following relevant co-interventions and confounding domains:

**Bias due to confounding**
Demographics and comorbidities participants
If PET-CT is more likely to be performed in participants with certain demographic characteristics and comorbidities that are associated with outcomes this could lead to a biased estimate of the effect of PET-CT.

Severity of disease
*Staphylococcus aureus* bacteremia is a heterogeneous disease and its clinical course ranges from very mild to extremely severe. If PET-CT was only performed in participants with severe disease, e.g. to diagnose metastatic sites in high-risk patients, this could lead to a bias towards PET being harmful with respect to mortality. On the contrary, if PET-CT was only performed in participants with mild disease, e.g. because patients with severe disease are too sick to undergo PET-CT, this could lead to a bias towards PET being beneficial.

Co-interventions
Clinical management of *Staphylococcus aureus* bacteremia consists of a complex interplay between multiple diagnostic (e.g. blood cultures, echocardiography) and therapeutic (e.g. source control interventions, antibiotic treatment) interventions. Protocols to guide clinical management are likely determined by local expertise, local protocols and period in time. Therefore, studies that were performed in multiple centers or used historical controls were considered at risk of bias.

**Bias in selection of participants into the study**Studies were required to describe how patients were included in and excluded from into the study and predefined subgroup analyses, if applicable.
Inclusion of participants that experienced outcomes before PET-CT could be performed could lead to immortal time bias. Studies were required to describe how they avoided this or adjusted for this.
 **Bias in classification of intervention**Studies were required to describe the length of time between diagnosis of *Staphylococcus aureus* bacteremia and performance of PET-CT. Studies with a wide range of time length were considered at risk of bias.

**Bias due to deviations from intended interventions**Variation in the procedures of preparation, execution and interpretation of the PET-CT, e.g. carbohydrate restriction, may affect outcomes. Studies were required to provide information on these topics.

**Bias due to missing data**Studies were required to include data from all participants in the primary analysis and describe how they handled missing data.

**Bias in measurement of the outcome**Studies were required to precisely define all outcomes they used in all analyses.

**Bias in selection of the reported result**Studies were required to report results as they defined in a prespecified protocol or in the study methods of the final report.
