# Supplementary material for: [18F]FDG-PET/CT in Staphylococcus aureus bacteremia: a systematic review
Source: BMC Infect Dis. 2022 Mar 24;22:282. doi: 10.1186/s12879-022-07273-x (PMC8943998; doi:10.1186/s12879-022-07273-x)
Supplement: Supplementary file 3 — Additional file 3. Table S1. Diagnostic findings on [18F]FDG-PET/CT per study. [file 12879_2022_7273_MOESM3_ESM.docx]

**Additional file 3: Table S1:** Diagnostic findings on [18F]FDG-PET/CT per study

| Study | **New diagnostic findings on [18F]FDG-PET/CT** |
| --- | --- |
| **Vos, 2010.** | NR |
| **Berrevoets, 2017.** | 165 metastatic foci of which 98 (59%) first detected by PET-CT in 74 (70%) of 105 SAB patients.  Vertebral osteomyelitis: 14 Arthritis or joint prosthesis: 19 Nonvertebral osteomyelitis: 10 Skin and soft tissue: 31 Psoas abscess: 7 Lung: 31 Spleen: 5 Liver/gallbladder: 3 Kidney: 1 Endocarditis: 18 Endovascular infection (excluding endocarditis): 21 Pericarditis or mediastinitis: 4 |
| **Berrevoets, 2019.** | **NR** |
| **Yildiz, 2019.** | 49 metastatic foci detected in 48 patients in the PET-CT group. 13 metastatic foci detected in 54 patients in the control group.  Spondylodiscitis: 12 in PET-CT group, 3 in control group Arthritis, joint prosthesis infection and/or osteitis: 16 in PET-CT group, 6 in control group Endovascular infection (excluding endocarditis): 3 in PET-CT group, 1 in control group Kidney: 1 in PET-CT group, 0 in control group Liver: 1 in PET-CT group, 0 in control group Lung: 5 in PET-CT group, 0 in control group Psoas abscess: 5 in PET-CT group, 2 in control group Sternocleidomastoid muscle: 3 in PET-CT group, 0 in control group Gluteal muscle: 1 in PET-CT group, 1 in control group Endocarditis: 1 in PET-CT group, 0 in control group Mediastinitis: 1 in PET-CT group, 0 in control group |
| **Ghanem-Zoubi, 2020** | New findings first detected by PET in 107 (71%) of 151 episodes.  Bone and joint: 58 Skin and soft tissue: 35 Muscles: 16 Vascular-extracardiac: 20 Cardiac: 9 Internal organs (abdomen, pelvis, thorax): 9 Lungs: 32 |

**NR, not reported; PET-CT, [18F]FDG-PET/CT.**
