# Supplementary material for: [18F]FDG-PET/CT in Staphylococcus aureus bacteremia: a systematic review
Source: BMC Infect Dis. 2022 Mar 24;22:282. doi: 10.1186/s12879-022-07273-x (PMC8943998; doi:10.1186/s12879-022-07273-x)
Supplement: Supplementary file 4 — Additional file 4. Appendix SC. Results risk of bias assessment individual studies with ROBINS-I. [file 12879_2022_7273_MOESM4_ESM.docx]

**Additional file 4: Appendix SC: Results risk of bias assessment individual studies with ROBINS-I.**

**Vos, 2010.**

| **Risk of bias domain** | **Outcomes assessed** | **Conclusion** | **Comment** |
| --- | --- | --- | --- |
| **Bias due to confounding** | 3-month infection relapse, 3-month mortality | Serious | For every prospectively included patient, 2 control patients were matched. 89% of study participants were perfectly matched. Matching performed on additional risk factors for presence of complicating infectious foci. No matching on age or comorbidity. Study was monocenter, but used historical controls. However, during the study period diagnostic work-up and treatment remained unchanged. Not reported how successful matching was for SAB patients specifically. |
| **Bias in selection of participants** | 3-month infection relapse, 3-month mortality | Moderate | It is clear how patients were included in the study. 2 patients in the study group died before a PET-CT was performed and 2 denied informed consent. These 4 patients were still included in the primary intention-to-treat analysis. |
| **Bias in classification of intervention** | 3-month infection relapse, 3-month mortality | Unknown | PET-CT performed after median of 7 days (mean 6.8d) after first positive blood culture became positive. Maximum time between first positive blood culture and PET-CT was 2 weeks. Ttime between SAB diagnosis and PET-CT not reported for SAB patients specifically. |
| **Bias due to deviations from intended interventions** | 3-month infection relapse, 3-month mortality | Moderate | Extensive information on preparation and execution of PET-CT. No information provided on definition of fasting prior to PET-CT. |
| **Bias due to missing data** | 3-month infection relapse, 3-month mortality | Unknown | Data of all patients, including those in whom PET-CT was not performed or who denied informed consent, were included in the primary analysis. No information on handling of missing data provided. |
| **Bias in measurement of the outcome** | 3-month infection relapse, 3-month mortality | Low | Outcomes were well defined. |
| **Bias in selection of the reported result** | 3-month infection relapse, 3-month mortality | Low | Study results reported as specified in methods. |

**Overall: Serious risk of bias.**

**Berrevoets, 2017.**

| **Risk of bias domain** | **Outcomes assessed** | **Conclusion** | **Comment** |
| --- | --- | --- | --- |
| **Bias due to confounding** | 3-month mortality, 3-month infection relapse in high risk SAB subgroup | Moderate | Multivariate analysis to adjust for potential confounders with 3-month mortality as outcome including composite risk score and Charlson Comorbidity Index. Subgroup analyses comparing PET-CT vs no PET-CT in patients with high-risk SAB. Study performed in one center during one time period. |
| **Bias in selection of participants** | 3-month mortality, 3-month infection relapse in high risk SAB subgroup | Moderate | Well described how participants were included in study.  Sensitivity analysis, excluding all patients that died within 7 days after admission, only performed with 3-month mortality as outcome. |
| **Bias in classification of intervention** | 3-month mortality, 3-month infection relapse in high risk SAB subgroup | Moderate | PET-CT was performed after a median of 8.0 and mean of 8.7 days. No measure of variance was reported. |
| **Bias due to deviations from intended interventions** | 3-month mortality, 3-month infection relapse in high risk SAB subgroup | Low | Extensive reporting on preparation for and execution of PET-CT. |
| **Bias due to missing data** | 3-month mortality, 3-month infection relapse in high risk SAB subgroup | Moderate | Data from all patients were included in the primary analysis.5 patients were lost to follow-up who were excluded. |
| **Bias in measurement of the outcome** | 3-month mortality, 3-month infection relapse in high risk SAB subgroup | Low | Outcomes were defined precisely. |
| **Bias in selection of the reported result** | 3-month mortality, 3-month infection relapse in high risk SAB subgroup | Moderate | Methods specify that cause of death was an outcome, but this is not reported in the results. |

**Overall: Moderate risk of bias.**

**Berrevoets, 2019.**

| **Risk of bias domain** | **Outcomes assessed** | **Conclusion** | **Comment** |
| --- | --- | --- | --- |
| **Bias due to confounding** | 3-month mortality, 3-month SAB specific mortality, 3-month infection relapse | Serious | No adjustment was performed for demographics, comorbidities and severity of disease of patients. Study was performed in two different centers.  Intervention and control group were included in the same time period. |
| **Bias in selection of participants** | 3-month mortality, 3-month SAB specific mortality, 3-month infection relapse | Moderate | Well described how participants were included in the study. No subgroup analyses were performed. No adjustment was performed for immortal time bias. |
| **Bias in classification of intervention** | 3-month mortality, 3-month SAB specific mortality, 3-month infection relapse | Moderate | FDG-PET/CT was performed after a mean of 8.4 days (SD 3.8 D). |
| **Bias due to deviations from intended interventions** | 3-month mortality, 3-month SAB specific mortality, 3-month infection relapse | Low | Intervention is well defined in methods. |
| **Bias due to missing data** | 3-month mortality, 3-month SAB specific mortality, 3-month infection relapse | Serious | 12 patients without follow-up after 3 months were excluded from the study population, which could have biased the outcomes. |
| **Bias in measurement of the outcome** | 3-month mortality, 3-month SAB specific mortality, 3-month infection relapse | Low | All outcomes were well defined in the methods. |
| **Bias in selection of the reported result** | 3-month mortality, 3-month SAB specific mortality, 3-month infection relapse | Serious | Results were reported as in study methods of the final report.  Incorrect conclusion that absence of a statistically significant difference between cases and control group means that both group have equal outcomes. |

**Overall: Serious risk of bias.**

**Yildiz, 2019.**

| **Risk of bias domain** | **Outcomes assessed** | **Conclusion** | **Comment** |
| --- | --- | --- | --- |
| **Bias due to confounding** | 1-month mortality, 3-month mortality, one-year mortality, new diagnostic findings related to SAB | Moderate | Univariate linear regression to evaluate association of variables and mortality. Selection of variables with p<0.05 with forward selection in multiple linear regression. Final model of association between performance of PET-CT and 30-day/90-day/1-year survival included kidney failure (eGFR<60 ml/min), diabetic foot infection, bacteremia of unknown origin, prosthetic or plate joint infection, septic arthritis and age<70 years. The study was executed in one hospital. Intervention and control group were included in the same time period. |
| **Bias in selection of participants** | 1-month mortality, 3-month mortality, one-year mortality, new diagnostic findings related to SAB | Serious | Well described how participants were included in study. The subgroup analyses of mortality in different age groups were not specified in the methods. No adjustment was performed for immortal time bias. |
| **Bias in classification of intervention** | 1-month mortality, 3-month mortality, one-year mortality, new diagnostic findings related to SAB | Serious | All 18-FDG PET/CT’s were performed within 1 week. No range of length of time between diagnosis of *Staphylococcus aureus* bacteremia and performance of 18-FDG PET-CT was provided. |
| **Bias due to deviations from intended interventions** | 1-month mortality, 3-month mortality, one-year mortality, new diagnostic findings related to SAB | Unknown | No information is provided on preparation and execution of 18-FDG PET/CT. All results of 18-FDG PET/CT were reviewed by two nuclear medicine physicians and one radiologist. |
| **Bias due to missing data** | 1-month mortality, 3-month mortality, one-year mortality, new diagnostic findings related to SAB | Unknown | Data of all participants was provided in the primary analysis. No information on handling of missing data was provided. |
| **Bias in measurement of the outcome** | 1-month mortality, 3-month mortality, one-year mortality, new diagnostic findings related to SAB | Low | Outcomes were well defined in the methods. |
| **Bias in selection of the reported result** | 1-month mortality, 3-month mortality, one-year mortality, new diagnostic findings related to SAB | Serious | No absolute or relative estimates of 30-day and 90-day mortality are provided, only p-values. |

**Overall: Serious risk of bias.**

**Ghanem-Zoubi, 2020.**

| **Risk of bias domain** | **Outcomes assessed** | **Conclusion** | **Comment** |
| --- | --- | --- | --- |
| **Bias due to confounding** | 1-month mortality, 3-month mortality, 6-month mortality, 6-month infection relapse, any intervention performed after bacteremia, duration of appropriate antibiotic treatment | Moderate | Controls were matched by age, Charlson score, methicillin susceptibility and survival duration to FDG-PET/CT. Residual confounding was controlled through regression analyses, including infection characteristics and interventions. The study was executed in one hospital and the inclusion period of the control group overlaps with the inclusion period of the intervention group. |
| **Bias in selection of participants** | 1-month mortality, 3-month mortality, 6-month mortality, 6-month infection relapse, any intervention performed after bacteremia, duration of appropriate antibiotic treatment | Moderate | Well described how participants were included in study and subgroup analyses. Immortal time bias was avoided by matching on survival duration to FDG-PET/CT and exclusion of patients with survival expectation of less than 1 week. |
| **Bias in classification of intervention** | 1-month mortality, 3-month mortality, 6-month mortality, 6-month infection relapse, any intervention performed after bacteremia, duration of appropriate antibiotic treatment | Low | FDG-PET-CT performed after 8-13 days after diagnosis with a median of 11 days. |
| **Bias due to deviations from intended interventions** | 1-month mortality, 3-month mortality, 6-month mortality, 6-month infection relapse, any intervention performed after bacteremia, duration of appropriate antibiotic treatment | Low | Intervention is well defined in methods. |
| **Bias due to missing data** | 1-month mortality, 3-month mortality, 6-month mortality, 6-month infection relapse, any intervention performed after bacteremia, duration of appropriate antibiotic treatment | Unknown | Outcomes of all patients are reported. Mortality data postdischarge updated in hospital’s health records from national registry. No information on handling of missing data was provided. |
| **Bias in measurement of the outcome** | 1-month mortality, 3-month mortality, 6-month mortality, 6-month infection relapse, any intervention performed after bacteremia, duration of appropriate antibiotic treatment | Low | Outcomes well defined in the methods. |
| **Bias in selection of the reported result** | 1-month mortality, 3-month mortality, 6-month mortality, 6-month infection relapse, any intervention performed after bacteremia, duration of appropriate antibiotic treatment | Moderate | Subgroup analyses were post hoc defined. Results were reported as in study methods of the final report. |

**Overall: Moderate risk of bias.**
